# Supplementary material for: Spatially Mapping the Baseline and Bisphenol-A Exposed Daphnia magna Lipidome Using Desorption Electrospray Ionization—Mass Spectrometry
Source: Metabolites. 2022 Jan 1;12(1):33. doi: 10.3390/metabo12010033 (PMC8781255; doi:10.3390/metabo12010033)
Supplement: Supplementary file 1 [file metabolites-12-00033-s001.zip › metabolites-1511709-supplementary.pdf]

Article

Spatially mapping the baseline and bisphenol-A exposed *Daphnia magna* lipidome using desorption electrospray ionisation - mass spectrometry

Matthew J. Smith [1], Ralf J. M. Weber [1] and Mark R. Viant [1,*]

[1] School of Biosciences, University of Birmingham, Edgbaston, Birmingham B15 2TT, UK; m.smith.15@bham.ac.uk (M.J.S.); r.j.weber@bham.ac.uk (R.J.M.W.)

* Correspondence: m.viant@bham.ac.uk

Supplementary methods

Preparing the cryogens

LN2 was decanted into a dewar for direct cooling of embedded samples and during optimisation of the snap-freezing process was used for preparation of cooled isopentane and condensed propane. Cooled isopentane was prepared by pouring isopentane into a copper cup and lowering into the LN2 until frozen. Upon removing the isopentane from the LN2 it began to thaw - creating a liquid to submerge the sample in. Condensed propane was prepared by capturing propane gas in a falcon tube and submerging the falcon tube in LN2 until the propane condensed ready for snap-freezing.

Supplementary Figures

Figure S1 - Bar chart summarising the quality of H&E stained *Daphnia magna* sections embedded in both 1% CMC + 9% gelatin and OCT embedding media; and frozen in LN2, LN2 cooled isopentane and LN2 cooled propane.

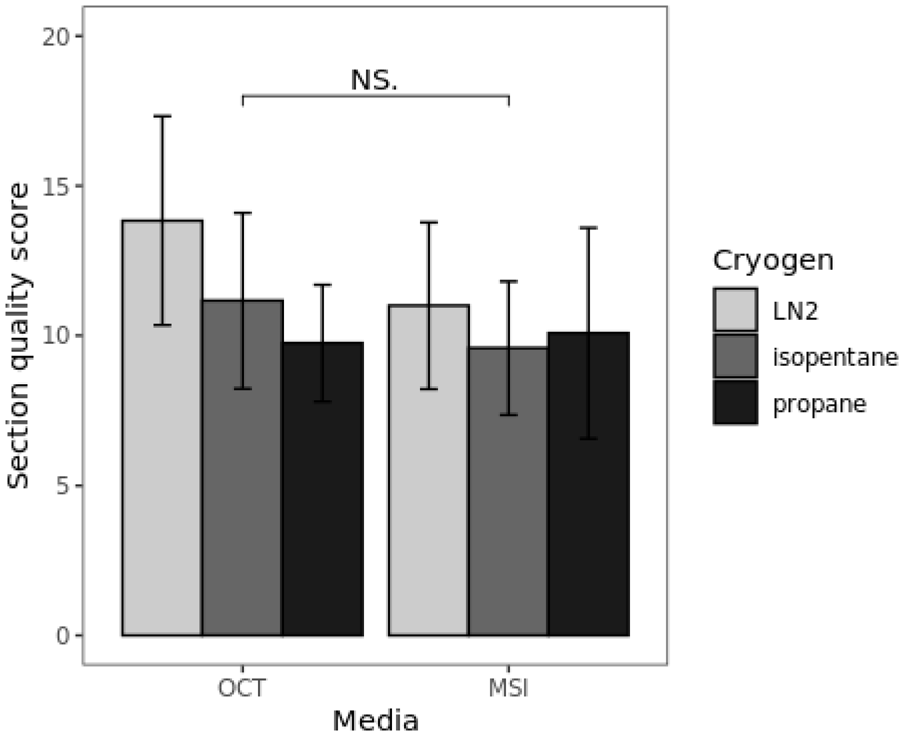

Figure S2 - Mass spectrum of polyalanine film following DESI-MS analysis of polyalanine film in positive ionisation mode.

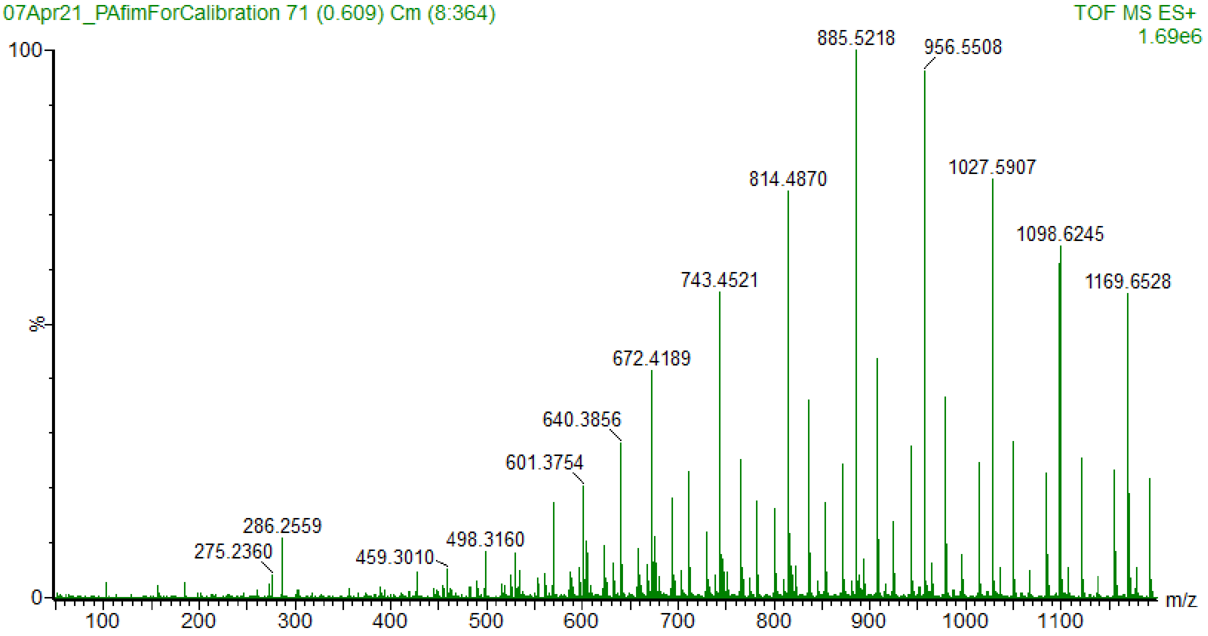

Figure S3 - Mass spectrum of porcine liver section following DESI-MS analysis in positive ionisation mode. Lipid profile between *m/z* 700 and 900.

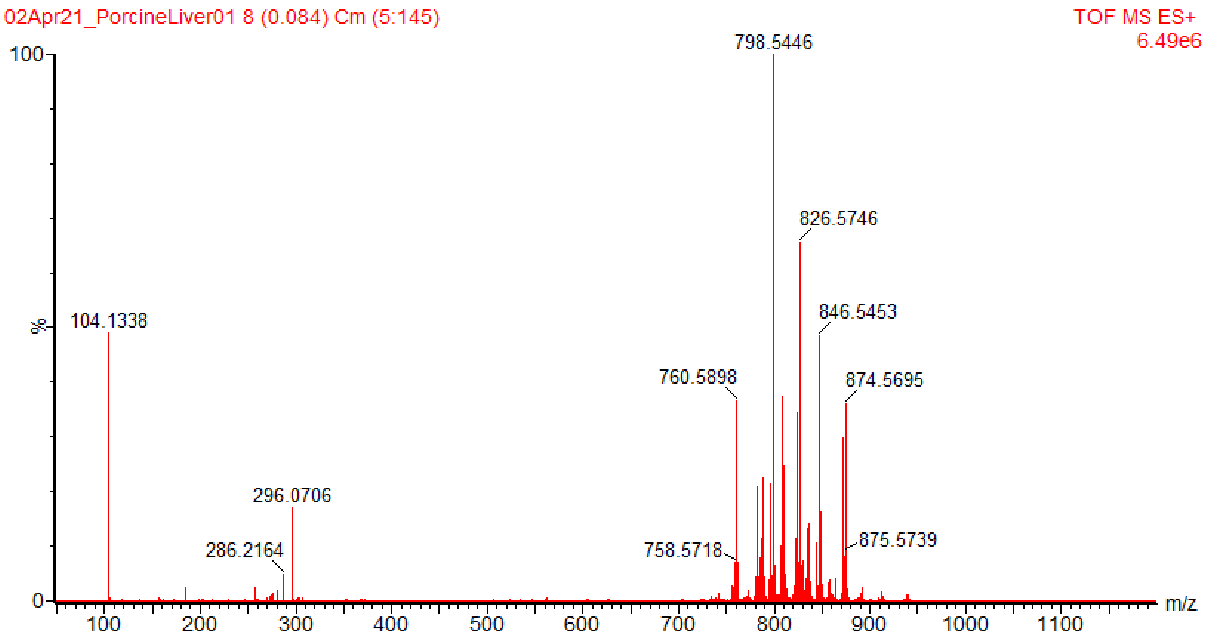

Figure S4 - Screenshots of process to extract only pixels pertaining to *Daphnia magna* tissue in HDImaging. (a) is the TIC image of the daphnid; (b) is the user defined ellipse tool to extract and (c) is the mask generated.

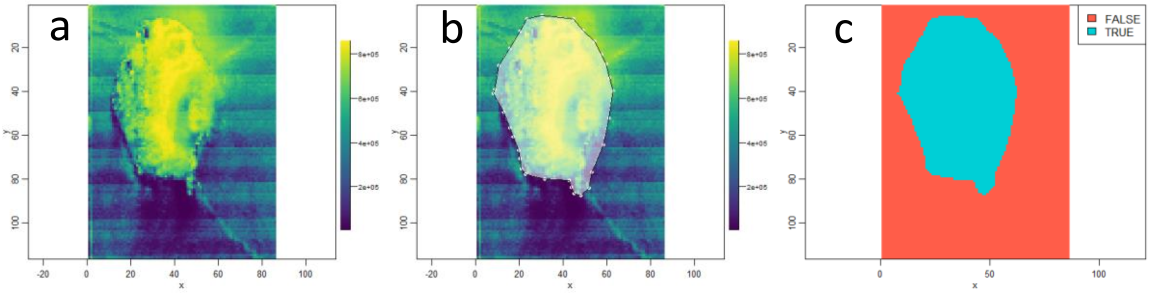

Figure S5 - PCA loadings plot from the DESI-MS analysis of 4 distinct tissues types in *Daphnia magna* sections - appendages, egg, eye and gut. Red points indicate pixels outside a 95% ellipse presumed to be important for distinguishing tissue types. Labels represent the putatively annotated lipid where possible or m/z value of each feature.

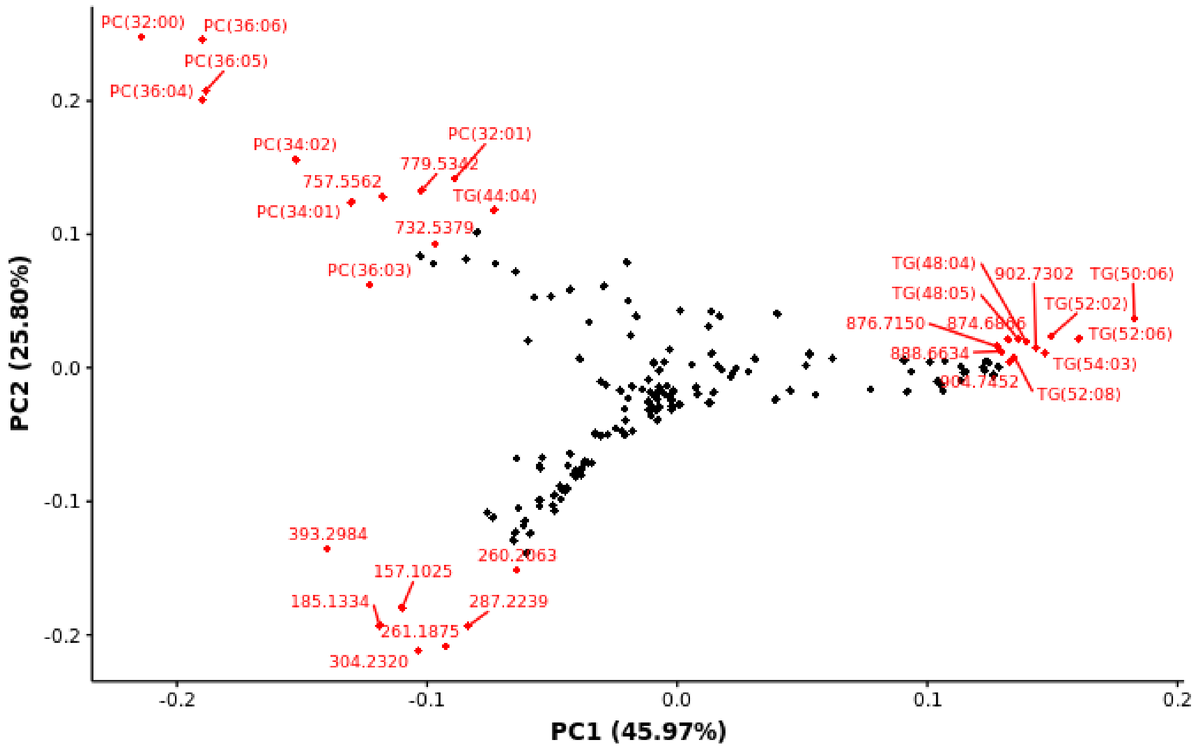

Figure S6 - PCA loadings plot from the DESI-MS analysis of *Daphnia magna* eggs across the 7th adult instar - specifically 8, 24, 48 and 72 h after the 6th molt. Red points indicate pixels outside a 95% ellipse presumed to be important for distinguishing tissue types. Labels represent the putatively annotated lipid where possible or m/z value of each feature. Time axis added as derived from the corresponding scores plot in Figure 5.

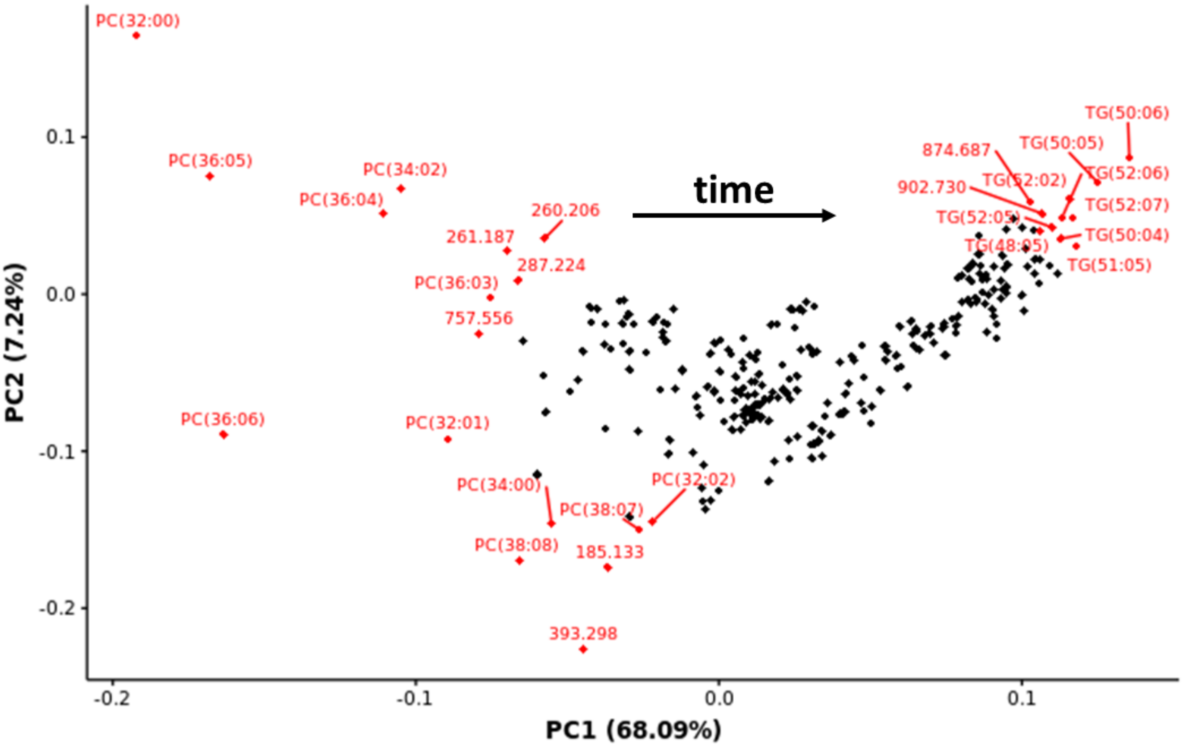

Figure S7 - Plots showing the percentage immobilisation after 72 h of exposure against log concentration of BPA - the lower plot is a zoomed version of the x-axis. From this plot the EC50 was calculated as 14 mg/kg.

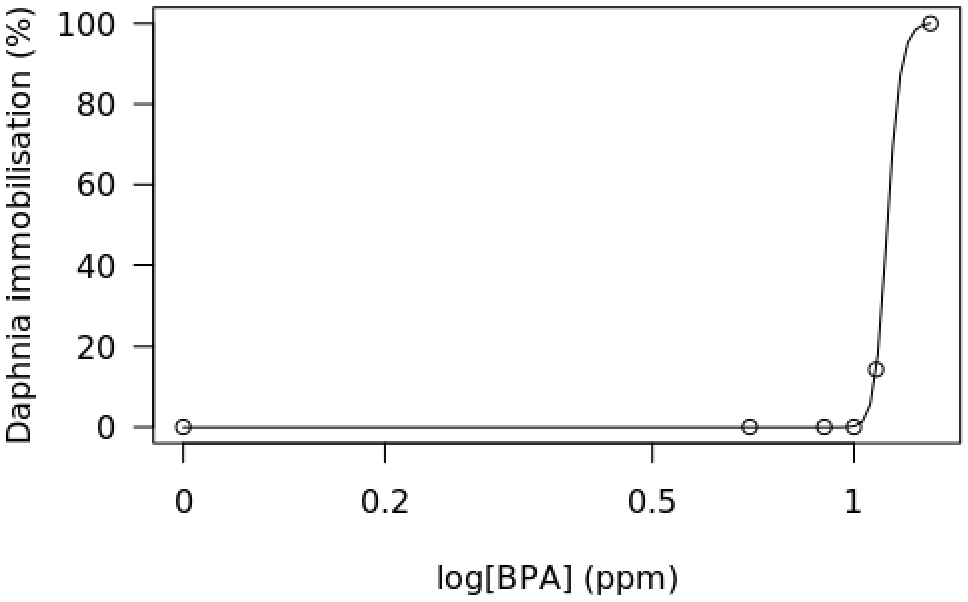

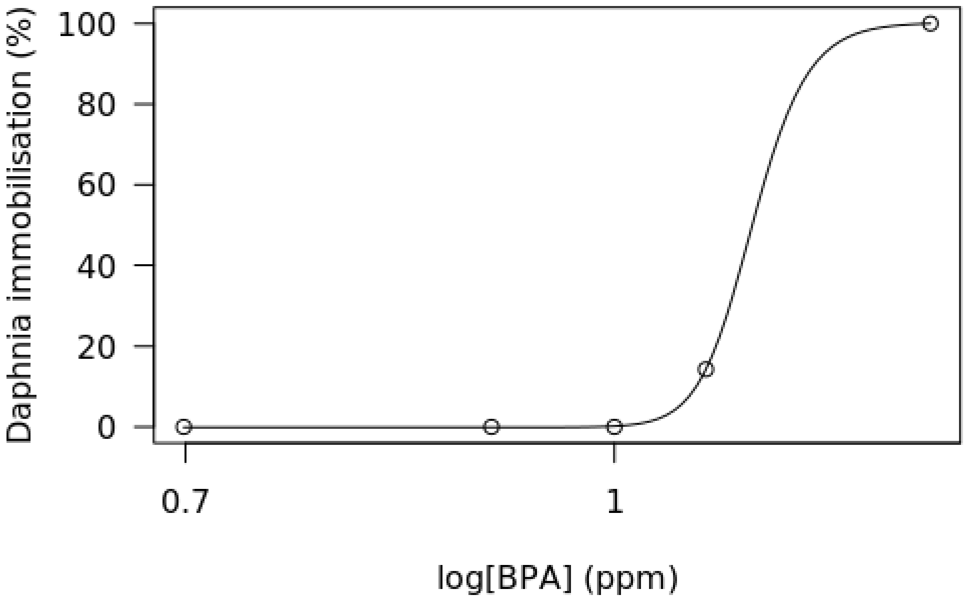

Figure S8 - PCA scores plot from the DESI-MS analysis of control *Daphnia magna* eggs and those exposed to 5 mg/kg bisphenol-A; (a) 8, (b) 24, (c) 48 and (d) 72 h into the 7th adult instar.

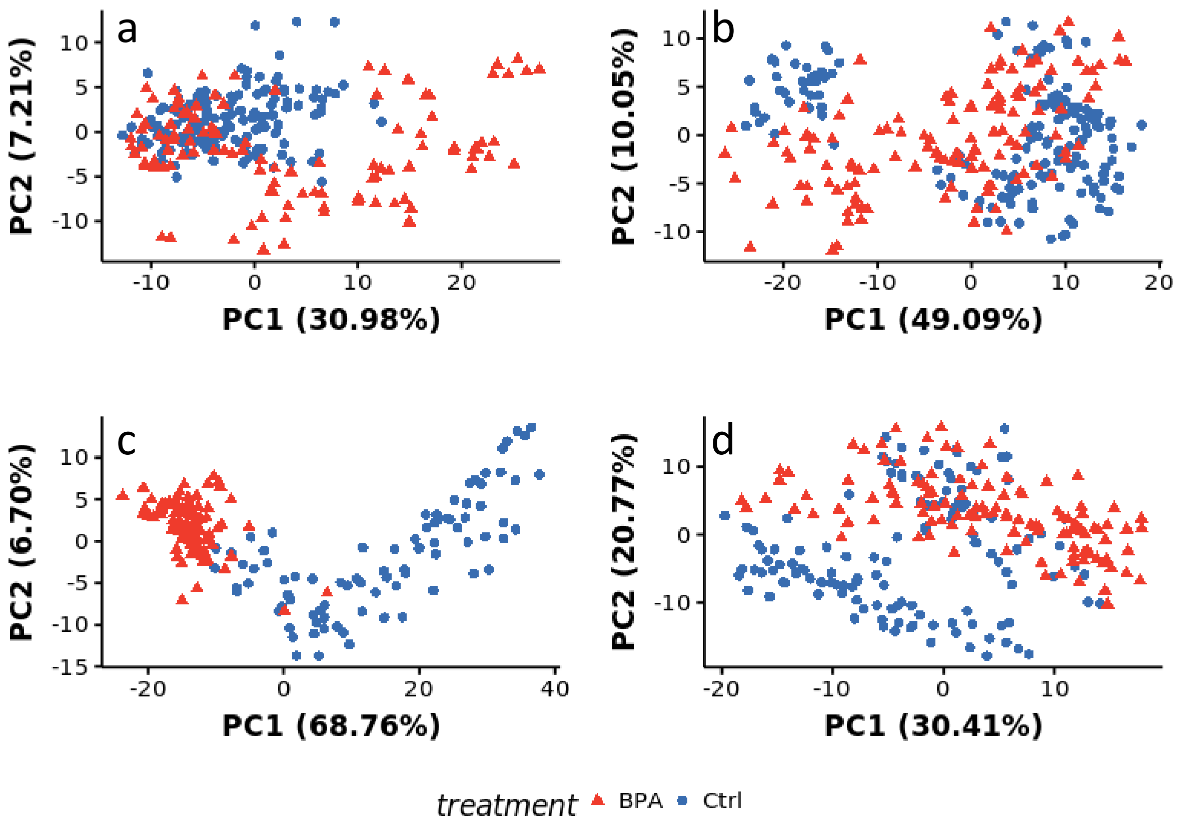

Figure S9 - PCA scores plot from the DESI-MS analysis of control *Daphnia magna* eggs and those exposed to 5 mg/kg bisphenol-A at 48 h. Each colour represents a pixel from a different animal, either treatment group or replicate it was taken from - highlighting the heterogeneity between eggs from the same daphnid and different replicates.

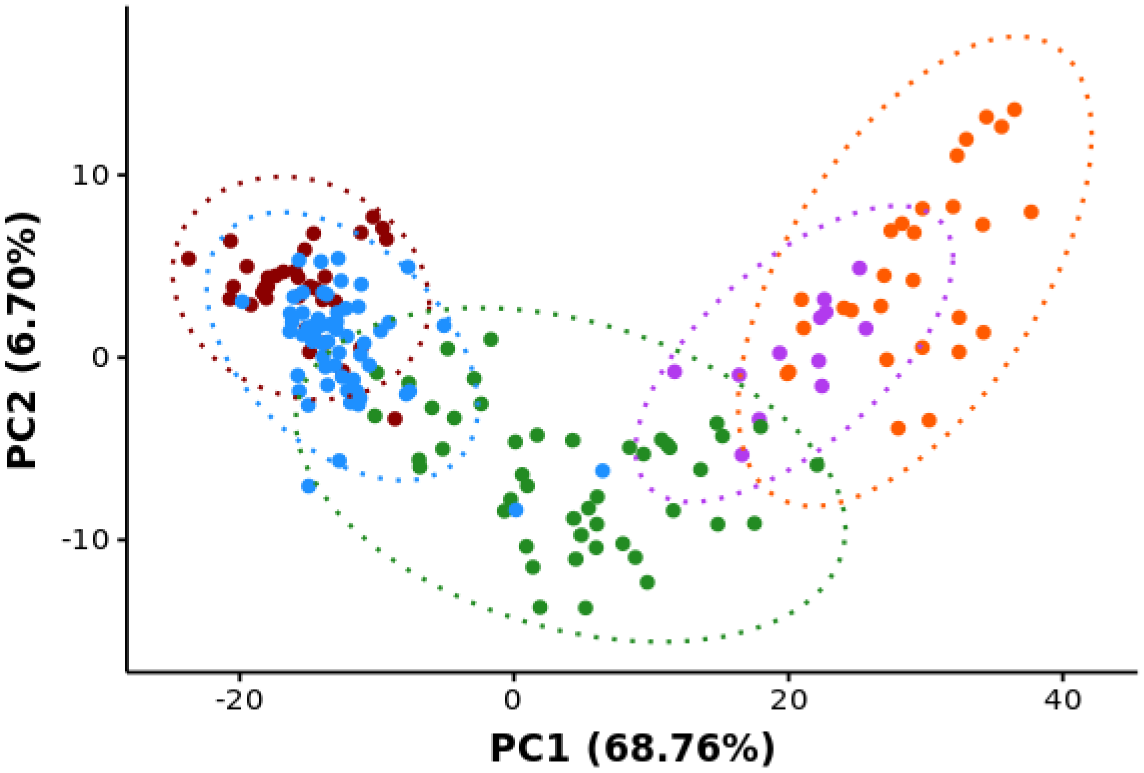

Figure S10 - PCA loadings plot from the DESI-MS analysis of control *Daphnia magna* eggs and those exposed to 5 mg/kg bisphenol-A at 48 h. Red points indicate pixels outside a 95% ellipse presumed to be important for distinguishing tissue types. Labels represent the putatively annotated lipid where possible or m/z value of each feature.

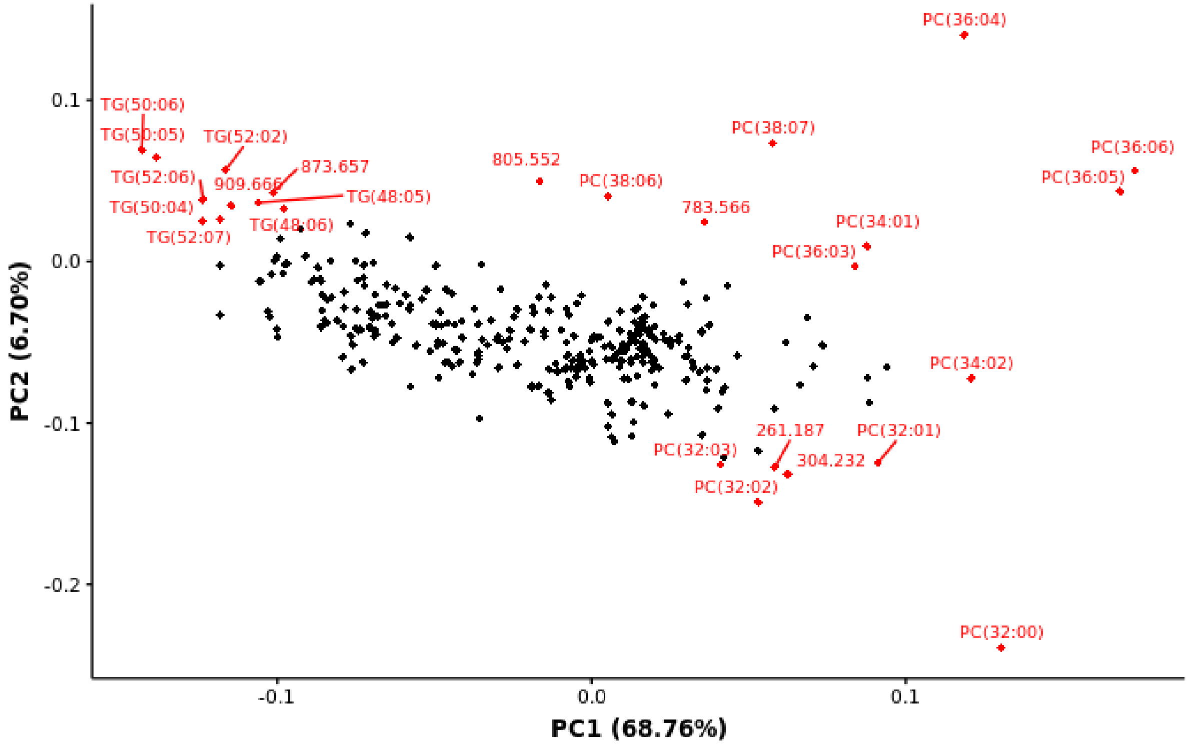

Supplementary Tables

Table S1 - Qualitative summary of embedding media formulations focusing on physical state at ambient temperature and cutting temperature as well as visual inspection of sections cooled in LN2. Pliable physical state refers to a smooth cutting surface which retains its structure without disintegrating upon sectioning (the gold standard). Other terms follow standard physical definitions.

Formulation	Physical state at ambient temperature	Physical state at cutting temperature	Sectioning quality	MSI compatible
OCT media	Fluid	Pliable	Good	No
Water	Fluid	Hard	Sample disintegrated upon sectioning	Yes
5% CMC	Viscous	Hard	Sample disintegrated upon sectioning	Yes
5% CMC + 5% gelatin	Viscous	Pliable	Cracks and holes visible	Yes
5% CMC + 10% gelatin	Viscous	Soft	Difficulty sectioning	Yes
2.5% CMC + 5% gelatin	Semi viscous	Semi-hard	Cracks and holes visible	Yes
2% CMC + 10% gelatin	Viscous	Pliable	Cracks and holes visible	Yes
1.5% CMC + 8% gelatin	Semi viscous	Pliable	Adequate	Yes
1% CMC + 9% gelatin	Semi fluid	Semi-hard	Adequate	Yes
1.5% agarose + 8% gelatin	Fluid	Semi-hard	Lateral lines visible	Yes
10% gelatin	Fluid	Semi-hard	Lateral lines visible	YES

Table S2 - Quantitative scoring system for assessing the quality of H&E stained Daphnia magna sections embedded in OCT media and frozen in LN2, LN2 cooled isopentane and LN2 cooled propane.

**Anatomy**	**LN2**						**Cooled isopentane**						**Cooled propane**					
	**D1**	**D2**	**D3**	**D4**	**D5**	**D6**	**D1**	**D2**	**D3**	**D4**	**D5**	**D6**	**D1**	**D2**	**D3**	**D4**	**D5**	**D6**
Eye (/2)	0	2	2	2	0	2	1	0	0	2	2	1	0	1	2	2	0	0
Gut (/5)	3	3	5	3	3	4	4	2	4	1	5	3	3	2	4	1	4	3
Eggs (/4)	1	4	4	1	2	2	2	2	1	2	2	2	2	0	1	2	2	1
Rostrum (/3)	2	3	3	3	1	1	3	1	0	3	2	1	1		2	2	1	2
Appendages (/3)	3	1	2	2	1	3	1	3	1	2	1	1	2	2	1	2	2	3
Overall outline (/3)	2	2	3	3	2	3	3	2	1	3	2	1	2	1	1	2	0.5	2
Total	11	15	19	14	9	15	14	10	7	13	14	9	10	6	11	11	9.5	11
**mean**	13.83333333						11.16666667						9.75					
**SDEV**	3.488074923						2.786873995						1.497219645					
**CV**	0.252149994						0.235510479						0.156231615					

Table S3 - Quantitative scoring system for assessing the quality of H&E stained Daphnia magna sections embedded in 1% CMC + 9% gelatin embedding media and frozen in LN2, LN2 cooled isopentane and LN2 cooled propane.

**Anatomy**	**LN2**						**Cooled isopentane**						**Cooled propane**					
	**D1**	**D2**	**D3**	**D4**	**D5**	**D6**	**D1**	**D2**	**D3**	**D4**	**D5**	**D6**	**D1**	**D2**	**D3**	**D4**	**D5**	**D6**
Eye (/2)	2	0	0	2	2	2	0	0	2	0	2	2	0.5	0	0	0.5	2	2
Gut (/5)	3	2	3	4	3	3	4	0.5	0.5	2	2	0.5	1	2.5	2.5	5	4	3
Eggs (/4)	1	0	1	3	1.5	1	1.5	2	2	3	3	2	2	1	2	3	1.5	1
Rostrum (/3)	2	1	1	2	1.5	2	1	0.5	1.5	1	2	3	2	0	0	1.5	1	2
Appendages (/3)	1	2	1.5	1.5	2	1.5	1.5	2	1.5	2	1.5	2.5	2.5	1	0.5	1	2.5	1
Overall outline (/3)	2	2	2.5	2.5	2.5	2	1.5	1	2	1.5	2.5	2	2.5	1	1	2.5	2	2
**Total**	11	7	9	15	12.5	11.5	9.5	6	9.5	9.5	10	13	12	10.5	5.5	6	13.5	13
**mean**	11						9.583333333						10.08333333					
**SDEV**	2.774887385						2.22298598						3.513070831					
**CV**	0.252262489						0.231963754						0.348403718					
